# Supplementary material for: Respectfulness-processing revisited: An ERP study of Chinese sentence reading
Source: PLoS One. 2022 Jun 24;17(6):e0258570. doi: 10.1371/journal.pone.0258570 (PMC9231749; doi:10.1371/journal.pone.0258570)
Supplement: S1 Table — (DOCX) [file pone.0258570.s001.DOCX]

**Table S1.** Examples of materials structure, including critical sentences and fillers, and the number of sentences of each type.

|  | **Type** | **Condition** | **Number** | **Sample sentence** |
| --- | --- | --- | --- | --- |
| Critical | c1d1 | Control | 40 | 林同学/对/张教授/说: “/您的/论文/我/已经/收到了。”/  Gloss: Student Lin/ to/ Professor Zhang/ said: “/ nin-de, ‘your [respectful]’/ article/ I/ have received.”  Translation: Student Lin said to Professor Zhang that I have received your article. |
|  | c1d2 | Respect violation | 40 | 张教授/对/林同学/说： “/您的/论文/我/已经/收到了。”  Gloss: Professor Zhang/ to/ Student Lin / said: “/ nin-de, ‘your [respectful]’/ article/ I/ have received.”  Translation: Professor Zhang said to Student Lin that I have received your article. |
|  | c2d1 | Semantic violation | 40 | 张教授/对/林同学/说： “/你的/论文/我/已经/收到了。 ”  Gloss: Professor Zhang / to/ Student Lin / said: “/ ni-de, ‘your [plain]’/ article/ I/ have received.”  Translation: Professor Zhang said to Student Lin that I have received your article. |
|  | c2d2 | Double violation | 40 | 林同学/对/张教授/说： “/你的/论文/我/已经/收到了。 ”  Gloss: Student Lin / to/ Professor Zhang / said: “/ ni-de, ‘your [plain]’/ article/ I/ have received.”  Translation: Student Lin said to Professor Zhang that I have received your article. |
| Fillers | f1 | For counterbalanc  First pro | 40 | 儿子/对/妈妈/说: “/我的/球拍/教练/已经/修好了。”/  Gloss: Son/ to /mom /said:”/ my /racket /coach /already/ fixed.”/  Translation: The son said to his mom: “my racket has been fixed by the coach.” |
|  | f2 | For counterbalanc  Third pro | 40 | 王同学/对/顾教授/说: “/他的/饮料/我/刚刚/买好了。”/  Gloss: Student Wang/ to /Professor Gu/said:”/ his /beverage/I/just/ bought.”/  Translation: Student Wang said to Professor Gu : “I just bought his beverage.” |
|  | f3 | syntactic violation | 20 | 一名/运动员/昨天/在操场/参加了/一场/很/比赛。/  Gloss:An /athelete/ yesterday/on the playground /took part in/a/very/game./  Translation: An athelete took part in a very game on the playground yesterday. |
|  | f4 | syntactic violation | 20 | 一名/很/小朋友/昨天/上午/在河边/钓了/一条鱼。/  Gloss: A/very/child/yesterday /morning/by the river/caught/a fish./  Translation: A very child caught a fish by the river yeaterday morning. |
|  | f5 | correct | 20 | 女孩/和/妹妹/年末/在服装店/买了/裙子。/  Gloss: The girl/ and/ the sister/ year end/ in the clothes shop/bought/skirt./  Translation: The girl and sister bought a skirt at the clothing store at the end  of the year. |
|  | f6 | correct | 20 | 老板/昨晚/在超市/采购了/蔬菜/和/鸡蛋。/  Gloss: The boss/ last night/ in the supermarket/ bought/ vegetables/and/eggs./  Translation: The boss bought vegetables and eggs in the supermarket last night |
|  | f7 | semantic violation | 20 | 红火的/医生/刚才/在病房里/耐心地/安慰了/女孩。/  Gloss: Prosperous/ doctor/ just now/in the sickroom/patiently/comforted/the girl./  Translation: The prosperous doctor patiently comforted the girl in the sickroom  just now. |
|  | f8 | semantic violation | 20 | 刻苦的/杨曦/这学期/在学校/全面地/复习了/伙食。/  Gloss: Hardworking/ Yang/ this semester/comprehensively/reviewed/food./  Translation: The hardworking Yang has reviewed his food  comprehensively in school this semester. |
|  | f9 | correct/active/  ba | 20 | 清洁工/把/大厦的/窗户/全部/擦/了/一遍。/  Gloss: The dustman/ ba/ the edifice’s/ windows/ all/wiped/ le/ once. /  Translation: The dustman wiped all the windows of the edifice’s once. |
|  | f10 | correct/passive/bei | 20 | 她/桌上的/首饰/被/小偷/拿走了。  Gloss: Her/ table/ jewelry/ bei/ a thief/taken. /  Translation: The jewelry on her table was taken by a thief. |
